# Resolution of Thyroid Eye Disease-Related Diplopia Following Intentional Myopic Targeting With Unilateral Cataract Surgery

**DOI:** 10.7759/cureus.106518

**Published:** 2026-04-06

**Authors:** Nayla Alkhater, Ammar Al-Mahmood

**Affiliations:** 1 Ophthalmology, Royal Medical Services, King Hamad University Hospital (KHUH), Muharraq, BHR

**Keywords:** anisometropia, general ophthalmology, persistent diplopia, phacoemulsification cataract surgery, thyroid eye disease (ted)

## Abstract

This case report describes the functional resolution of symptomatic diplopia in a patient with stable thyroid eye disease (TED) following unilateral cataract surgery with intentional myopic refractive targeting.

A 58-year-old patient with inactive TED presented with persistent distance diplopia and a visually significant unilateral cataract. Prism correction was poorly tolerated, and the patient declined strabismus surgery. Cataract extraction with monofocal intraocular lens implantation was performed, intentionally targeting a postoperative refraction of −3.00 diopters to induce functional anisometropia. At the six-month follow-up, the achieved refraction was −2.25 diopters. The patient reported subjective resolution of diplopia at both distance and near, with improved binocular comfort. Ocular motility remained unchanged, supporting a functional rather than mechanical mechanism.

The aim of this case report is to highlight a novel diplopia treatment modality specifically for TED euthyroid patients. Intentional myopic refractive targeting during unilateral cataract surgery may represent a noninvasive functional option for alleviating diplopia in carefully selected patients with stable TED.

## Introduction

Diplopia is a common and often disabling manifestation of thyroid eye disease (TED), most frequently arising from restrictive extraocular muscle involvement. TED should always be a consideration in patients with unexplained diplopia [1]. TED, also known as Graves' orbitopathy, causes swollen extraocular muscles and orbital fat. Mechanistically, TED involves lid retraction, oedema and redness of the eyelids and conjunctiva, proptosis, diplopia, and optic neuropathy [2]. One-third of these suffer from constant diplopia [3]. Standard management options include prism correction, botulinum toxin injection, and strabismus surgery which was declined by our patient. Strabismus surgery should be carried out only after the inflammation is no longer active and after any decompression surgery. Surgery comprises the recession of tight muscles using adjustable sutures so as to maximize the area of binocular single vision [4]. However, these approaches may be poorly tolerated or declined by patients. 

This report describes intentional myopic refractive targeting during cataract surgery as a functional strategy to alleviate TED-related diplopia. Alteration of refractive status can influence binocular vision by modifying image clarity and fusion demand. Induced anisometropia has been shown to reduce binocular summation and facilitate the suppression of a diplopic image. Surgically induced anisometropia is well tolerated by some individuals, while others experience binocular visual complaints [5]. In the context of diplopia, induced anisometropia refers to a purposeful clinical approach in which a significant, intentional difference in refractive power (generally 2.0-3.0+ diopters) is generated between the two eyes, commonly through contact lenses or cataract surgery. This treatment aims to create a blur in one eye, which compels the brain to suppress the image of that eye and erase the distracting second image of diplopia.

## Case presentation

A 58-year-old patient with inactive TED (Clinical Activity Score=0 for over 12 months) and persistent diplopia was evaluated. Diplopia had been present for more than six months, predominantly during distance fixation, and interfered with daily activities. The patient was biochemically euthyroid.

**Figure 1:**
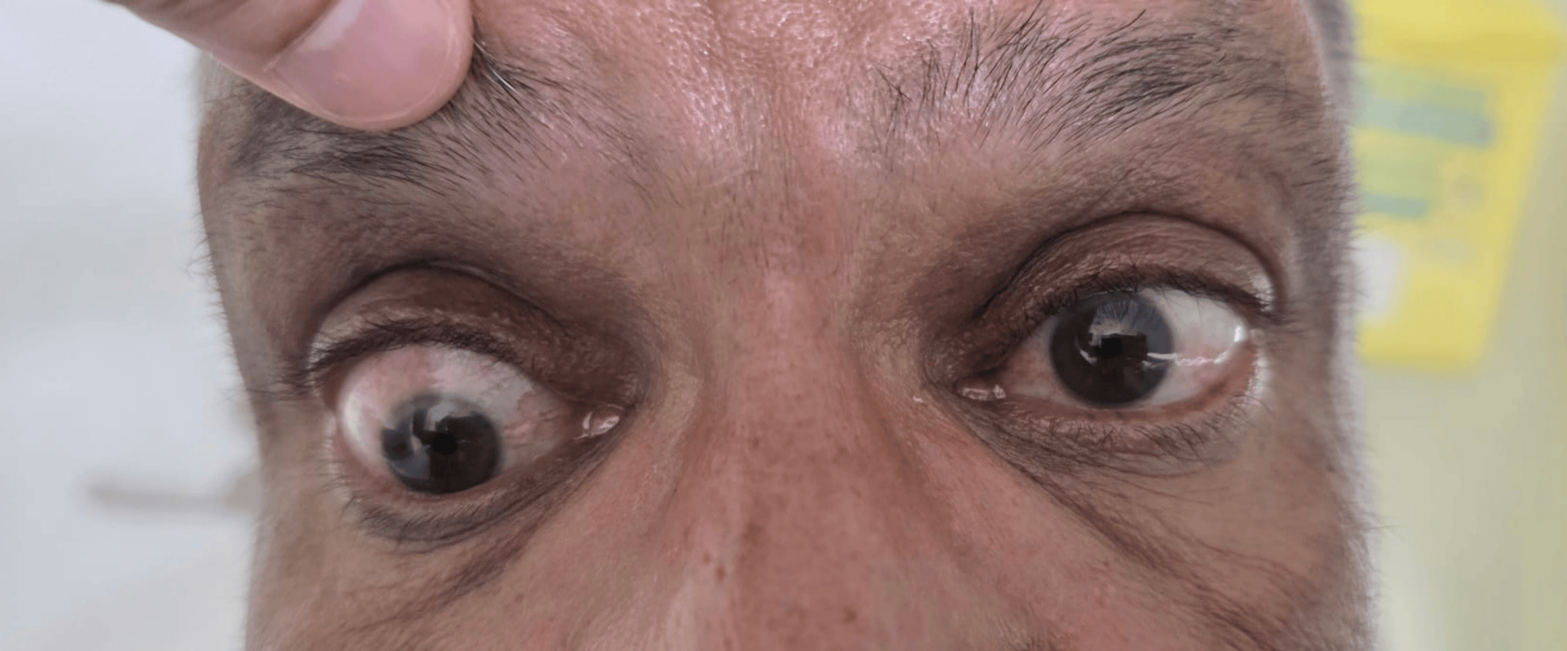
Patient in primary gaze showing vertical ocular misalignment consistent with a restrictive pattern of thyroid eye disease The affected eye (right eye) demonstrates hypotropia in the primary position, suggestive of inferior rectus restriction with mild periorbital fullness.

**Figure 2:**
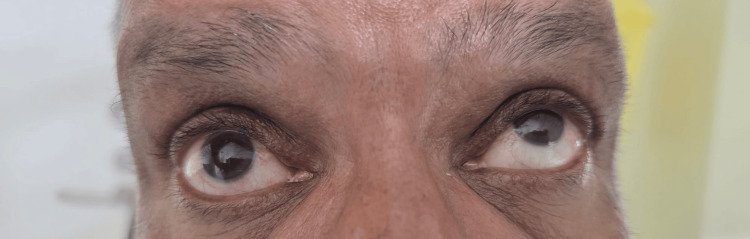
Patient in up-gaze demonstrating marked limitation of elevation of the affected eye with compensatory overaction of the fellow eye

Best-corrected visual acuity in the affected eye was 6/120 due to a dense nuclear sclerosis cataract with a posterior subcapsular component. The fellow eye had good visual acuity with a mild cataract. Ocular motility showed a mild restrictive pattern consistent with chronic TED. Eye movements are restricted due to edema that occurs in the extraocular muscles during the infiltrative stage and the subsequent fibrosis. Diplopia manifesting as the appearance of overlapping images is common. In primary and reading positions, it affects daily activities and causes patients significant discomfort [6] (Figures 1-2).

Prism correction was poorly tolerated, and the patient declined strabismus surgery. Given the presence of a unilateral visually significant cataract and stable motility disturbance, intentional anisometropia was planned by targeting a postoperative refraction of −3.00 diopters in the affected eye.

Surgical technique

**Figure 3:**
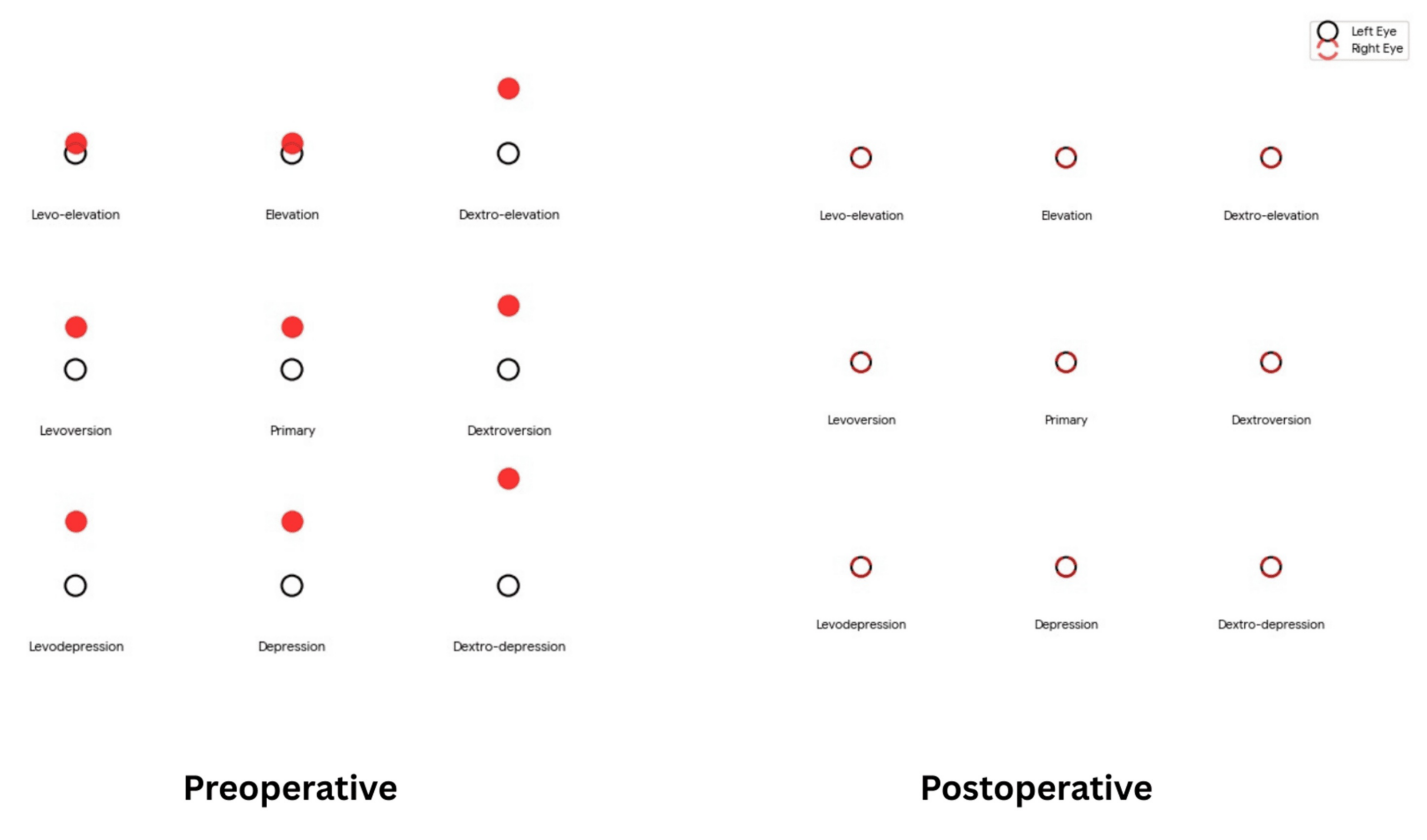
Before-and-after comparison of diplopia using a red glass test across the nine gaze positions Preoperative: Diplopia present (right inferior rectus overaction/restriction pattern). The red dot (right eye) and black dot (left eye) are separated in most gaze positions; this represents vertical diplopia (one image higher than the other). Postoperative: After diplopia resolved. The red and black dots are perfectly superimposed in all nine gaze positions, achieved by intentional anisometropia.

Standard phacoemulsification cataract surgery was performed without complication. Biometry calculations were deliberately adjusted to target −3.00 diopters using a monofocal intraocular lens. The postoperative course was uneventful. Figure 3 illustrates the diplopia chart which represents sensory orthophoria achieved with induced anisometropia while underlying restrictive strabismus remains. 

Outcomes

At one month postoperatively, uncorrected visual acuity was 6/20. Manifest refraction was −2.25 diopters. At six months, the patient reported subjective resolution of diplopia at distance and near, with improved binocular comfort. Ocular motility remained unchanged. The patient did not require prism correction or further surgical intervention. Table 1 summarizes the findings preoperatively and six months postoperatively. 

**Table 1:** Preoperative and postoperative findings of the resolution of thyroid eye disease-related diplopia six months following induced myopic targeting by cataract surgery This table illustrates the preoperative findings of a 58-year-old thyroid eye disease patient with persistent diplopia for more than six months, who underwent cataract surgery, compared with his postoperative outcomes, which show resolved diplopia and improved comfort. NS: nuclear sclerosis; PSC: posterior subcapsular cataract; VA: visual acuity; IR: inferior rectus; Phaco: phacoemulsification; IOL: intraocular lens

Parameter	Preoperative findings	Postoperative findings (6 months)
Thyroid status	Euthyroid	Stable
Diplopia	Persistent >6 months, distance >near	Resolved subjectively
VA (affected eye)	6/120 (dense NS + PSC)	6/20 (uncorrected)
Fellow eye	Good VA, mild cataract	Unchanged
Ocular motility	Restrictive, IR limitation, hypotropia	No change
Prism	Poor tolerance	Not required
Strabismus surgery	Declined	Not needed
Procedure	-	Phaco + monofocal IOL
Refractive target	Planned −3.00 D	Achieved −2.25 D
Binocular function	Diplopia, poor comfort	Improved comfort
Complications	None	None

## Discussion

Diplopia management in TED is challenging, particularly in patients with stable restrictive disease who are unable or unwilling to undergo strabismus surgery. Prism correction may be limited by intolerance or inadequate relief. In this case, refractive targeting provided a functional alternative by modifying sensory input rather than ocular alignment. TED‑related strabismus needs careful evaluation and management to achieve an optimal outcome. Different surgical and non‑surgical options are available for intervention in TED‑related strabismus [7].

Intentional anisometropia and pseudophakic monovision have previously been reported as strategies for managing long-standing diplopia in cataract patients. Like other studies, Hunter described extreme anisometropic pseudophakic monovision to suppress diplopia [8]. Similar approaches were reported by Snyder and Perez [9]. However, these studies did not focus specifically on TED. The present case extends this concept to inactive TED-related restrictive diplopia.

Myopic defocus facilitates the sensory suppression of the secondary diplopic picture by reducing binocular summation and distance image clarity. The visual system is better able to overlook the mismatched information when one of the competing images is of lower quality, which may result in a perceptual decrease in double vision. Simultaneously, the induced myopia may improve near visual function, providing a functional effect for close work or tasks like reading. In properly chosen patients, purposeful myopic targeting may be an effective strategy due to this dual effect. The unchanged ocular motility supports a sensory mechanism.

Limitations include the single-case design and reliance on subjective outcomes. During the past two decades, several studies have compared the outcome of strabismus surgery on patients with and without previous orbital decompression. Their results have varied; some found that patients with thyroid ophthalmopathy who underwent orbital decompression have a lower success rate of surgery for strabismus, whereas others contradicted them reporting that previous orbital decompression surgery had no significant effect on the characteristics or outcomes of strabismus surgery [10].

## Conclusions

Intentional myopic refractive targeting during unilateral cataract surgery may offer a noninvasive, patient-centered option for alleviating diplopia in selected patients with stable TED. This approach may eliminate the perception of double vision and enhance general visual function by intentionally inducing a degree of anisometropia, hence decreasing the need for more invasive surgical procedures. The use of intentional anisometropia for the treatment of diplopia related to TED, especially when accomplished by less invasive techniques, has not received sufficient attention in the literature to date, and the majority of published work has highlighted corrective methods such as strabismus surgery. In this context, using phacoemulsification with a specific refractive target is a relatively novel concept that highlights an evolution toward more less invasive conservative techniques that prioritize safety and functional visual improvement first. Moving forward to treat TED-related diplopia patients with the given technique and eventually studying the outcomes would give us an overview of the long-term prognosis and patient satisfaction. 
